# NF-κB Regulates Mesenchymal Transition for the Induction of Non-Small Cell Lung Cancer Initiating Cells

**DOI:** 10.1371/journal.pone.0068597

**Published:** 2013-07-30

**Authors:** Manish Kumar, David F. Allison, Natalya N. Baranova, J. Jacob Wamsley, Adam J. Katz, Stefan Bekiranov, David R. Jones, Marty W. Mayo

**Affiliations:** 1 Department of Biochemistry & Molecular Genetics, University of Virginia, Charlottesville, Virginia, United States of America; 2 Department of Surgery, University of Virginia, Charlottesville, Virginia, United States of America; 3 Division of Plastic and Reconstructive Surgery, Department of Surgery, University of Florida College of Medicine, Gainesville, Florida, United States of America; H. Lee Moffitt Cancer Center & Research Institute, United States of America

## Abstract

The epithelial-to-mesenchymal transition (EMT) is a de-differentiation process that has been implicated in metastasis and the generation of cancer initiating cells (CICs) in solid tumors. To examine EMT in non-small cell lung cancer (NSCLC), we utilized a three dimensional (3D) cell culture system in which cells were co-stimulated with tumor necrosis factor alpha (TNF) and transforming growth factor beta (TGFβ). NSCLC spheroid cultures display elevated expression of EMT master-switch transcription factors, *TWIST1*, *SNAI1/Snail1*, *SNAI2/Slug* and *ZEB2/Sip1*, and are highly invasive. Mesenchymal NSCLC cultures show CIC characteristics, displaying elevated expression of transcription factors *KLF4*, *SOX2*, *POU5F1/Oct4*, *MYCN*, and *KIT*. As a result, these putative CIC display a cancer “stem-like” phenotype by forming lung metastases under limiting cell dilution. The pleiotropic transcription factor, NF-κB, has been implicated in EMT and metastasis. Thus, we set out to develop a NSCLC model to further characterize the role of NF-κB activation in the development of CICs. Here, we demonstrate that induction of EMT in 3D cultures results in constitutive NF-κB activity. Furthermore, inhibition of NF-κB resulted in the loss of *TWIST1*, *SNAI2*, and *ZEB2* induction, and a failure of cells to invade and metastasize. Our work indicates that NF-κB is required for NSCLC metastasis, in part, by transcriptionally upregulating master-switch transcription factors required for EMT.

## Introduction

Cancer development from early pre-malignant neoplasm to full metastatic disease is a multistep process that involves tumor epithelial-stromal interactions, angiogenesis, and infiltration of tumor-associated pro-inflammatory cells [1], [2]. An emerging hypothesis proposes that this milieu of cell-cell interactions, growth factors, and cytokines known as the tumor microenvironment, stimulates morphogenesis within tumor cells referred to as the epithelial-to-mesenchymal transition (EMT) [3]–[5]. EMT induces a redistribution of intracellular architecture, decreased cell-cell adhesion, and loss of cellular polarization. Carcinoma cells that have undergone EMT are characteristically motile, invasive and highly metastatic. Over the past several years, EMT has also been recognized as a de-differentiation program attributed to generation of tumor-initiating or cancer-initiating cells (CICs) that are important in the maintenance of cancer “stemness” [6]–[9].

Although multiple cytokines and growth factors induce EMT, one of the best studied factors is transforming growth factor beta (TGFβ) [2], [3], [10]–[13]. Stimulation of cells with TGFβ results in expression of the EMT master-switch transcription factors, TWIST1, SNAI1/Snail, SNAI2/Slug, and ZEB2/Sip1 that together differentially regulate genes to promote the mesenchymal phenotype [10], [12]. While extensive research details the ability for TGFβ to induce EMT, evidence indicates that tumor necrosis factor (TNF) further potentiates the transition [14], [15]. During cancer progression, secretion of TGFβ within the tumor microenvironment occurs through many different cell types, including tumor-associated fibroblasts, while secretion of TNF originates from tumor-associated M2 macrophages [3], [16], [17]. A prevailing hypothesis in the field is that exposure of cancer cells to these cytokines within the tumor microenvironment promotes EMT, de-differentiation, and the formation of CICs [2], [5], [17].

TNF is a powerful pro-inflammatory cytokine that stimulates signaling cascades to activate nuclear factor kappa B (NF-κB). As a transcription factor, NF-κB plays a key role in the expression of genes involved in cancer initiation and progression. Upregulation of NF-κB activity often occurs in primary solid and hematological tumors, directly correlating with de-differentiated morphology, advanced tumor stage, and poor clinical prognosis [18]. Importantly, NF-κB has been linked to mammary CICs [19], [20]. NF-κB induces and maintains EMT in model systems through two mechanisms, upregulation of EMT master-switch transcription factors [21]–[24] and stabilization of Snail [25]. NF-κB is composed of five Rel family members: RelA/p65, RelB, cRel, p50 and p52. In unstimulated cells, inhibitory IκB subunits associate with NF-κB dimers and sequester them in the cytoplasm. Upon cellular stimulation by pro-inflammatory cytokines, IκBα is phosphorylated by the IκB kinase (IKK) complex, ubiquitinated by the SCF-type E3 ligase, E3RS^IκB/β-TrCP^ and degraded by the 26S proteasome [26]. Liberated NF-κB then translocates to the nucleus to activate gene expression by recruiting transcriptional coactivators [27]. Our laboratory has shown that posttranslational modifications on RelA are required for full NF-κB transcriptional activity [27]–[30].

Although EMT in breast cancer models requires NF-κB activity [31], the role of this transcription factor in stimulating EMT and developing CICs in NCSLC has not been thoroughly examined. However, strong evidence exists for the presence of NSCLC stem/progenitor cells in primary adenocarcinomas and established cell lines [32]–[35]. Here, we demonstrate that coordinated activation of TNF and TGFβ signaling cascades effectively induces EMT and the expression of genes related to de-differentiation and stemness. Further, we show that mesenchymal NSCLC cells possess constitutively active NF-κB, and that inhibition of NF-κB decreases EMT, CIC formation, and metastatic potential.

## Materials and Methods

### Cell culture and reagents

NSCLC lines A549, H359, H1299, and H157 were obtained from ATCC and maintained as 2D cultures in DMEM (CellGro), 10% FBS (Invitrogen) and penicillin/streptomycin (Invitrogen). The antibodies used include: E-cadherin (BD Pharmingen- 610404), N-cadherin (BD Pharmingen- 610920), Vimentin (V6630), Fibronectin (BD Pharmingen- 610078), α-Tubulin (Sigma T6793), HMGA2 (Biocheck 59170AP), Twist1 (Cell Signaling 4119), Snail1 (Cell Signaling 4719), Sip1 (SCBT sc-48789), Slug (Abcam ab27568), IκBα (pS32, Cell Signaling 2859), IκBα (SCBT sc-371), RelA (pS536, Cell Signaling 3031), RelA (SCBT sc-372), and M2-Flag (Sigma F1804). Baculogold protease inhibitors were obtained from BD Biosciences. TGFβ (PHG 9204) and TNF (PHG 3015) were purchased from Invitrogen/Life technologies. All other chemicals were from Sigma.

### Three-dimensional multicellular spheroid cultures

Three-dimensional multicellular spheroid cultures were created using a modified hanging droplet method [36]. Cells were grown to approximately 80% confluence on standard tissue-culture plates. The cells were subsequently trypsinized, resuspended in DMEM/10% FBS, and counted. To create 25,000 cell spheroids, the cell suspension was diluted to a concentration of 1,000,000 cells/ml, and 25 µl of the cell suspension were pipetted onto the underside of a sterile 10 cm tissue-culture plate lid. Each lid holds approximately fifty droplets. After loading the droplets, the lid was placed onto a tissue culture plate containing 6 mL of sterile PBS and incubated for 48 hours to facilitate cellular aggregation and spheroid formation. The freshly formed spheroids were then transferred into 10 cm suspension plates containing DMEM and 2% FBS to prevent cell attachment to the dish. Suspension plates were made by adding 8 ml of poly-HEMA solution (Sigma-Aldrich P3932, 10 mg/ml) in 95% ethanol to sterile polystyrene petri dish plates (Fisher Scientific). The plates were then incubated for 24 hours in a sterile environment to allow the ethanol to evaporate. Prior to use, plates were washed with sterile PBS to remove any residual ethanol or other contaminants. Each suspension plate holds up to 100 spheroids. After transfer, the spheroids were treated with vehicle or with 10 ng/ml TNF and 2 ng/ml TGFβ, and incubated for 48 hours. After incubation, cells were subjected to a second treatment of vehicle or TNF and TGFβ, and incubated an additional 48 hours. The spheroids were then collected and analyzed by various assays.

### Immunofluorescence Microscopy

A549 cells were seeded on glass coverslips and subjected to EMT induction or left untreated. After induction, the cells were fixed in 100% methanol and subsequently incubated with primary antibodies to the extracellular domain of E-Cadherin (SCBT, sc-7870). An AlexaFlour-conjugated, goat anti-rabbit antibody (Invitrogen) was used as a secondary antibody, and indirect immunofluorescence of E-Cadherin was imaged using a Nikon E3800 fluorescence microscope.

### Migration and Invasion

*In vitro* migration and invasion assays were carried out according to the manufacturer's protocol (BD Biosciences). 2D and 3D cultures were disaggregated by trypsin and subsequently counted. 1×10^5^ cells (migration) or 1×10^4^ cells (invasion) were seeded in plain DMEM in the top well of a transwell control plate (BD 354578) or Matrigel invasion plate (BD 354480). The bottom well was loaded with DMEM containing 10% FBS as a chemoattractant, and the plates were incubated for eight hours (migration) or twenty-four hours (invasion) at 37°C and 5% CO_2_. Afterwards, cells on the upper side of the membrane were removed, and the remaining cells were fixed in 100% methanol and stained with 0.1% crystal violet. The stained cells were imaged and quantified using Adobe® Photoshop.

### Tumor model

Monolayer (2D) and 3D A549 cultures that had been left untreated or treated with TNF and TGFβ were trypsinized, resuspended in DMEM/0.5% FBS, and carefully counted and diluted in the appropriate volume for injection. Cells were subcutaneously (SC) injected into female outbred Crl:NU/NU nude mice (Charles River). Five mice were injected per experimental condition. All animal studies were performed as three independent experiments. Mice were sacrificed forty days post-injection. The primary SC tumors were removed and weighed. Additionally, the lungs were removed, fixed in formalin, and surface lung metastases were counted. To quantify the amount of total tumor burden in the formalin fixed lung tissue, genomic DNA was extracted [37] and assayed for the presence of human genomic material as described using quantitative real time-polymerase chain reaction (QRT-PCR) primers specific to human endogenous retrovirus-3 (ERV3, Table S1) [38], [39].

This study was carried out in strict accordance with recommendation from the Animal Care and Use Committee (ACUC) of the University of Virginia. The protocol was approved by ACUC Number 3914. All experiments were terminated after 40 days at which time SC tumors were less than 1.0 cm^3^ in size; thus, restricting tumor burden. All efforts were made to minimize pain and suffering.

### QRT-PCR, Immunoblots, and Electrophoretic mobility shift assays (EMSAs)

QRT-PCR and immunoblot experiments were carried out as previously described [28]. PCR primers are shown in Table S1. Nuclear extracts were prepared using spheroids from A549.V and A549.I cell lines treated with or without TNF and TGFβ. EMSAs and supershift assays were performed as described previously [40].

### Statistics

Where appropriate, comparisons between experimental groups were carried out by performing a one-tailed Student's *t* test in Microsoft excel. Data for all experiments was considered statistically significant when p<0.05.

## Results

### A model to study EMT in NSCLC

**Figure 1:**
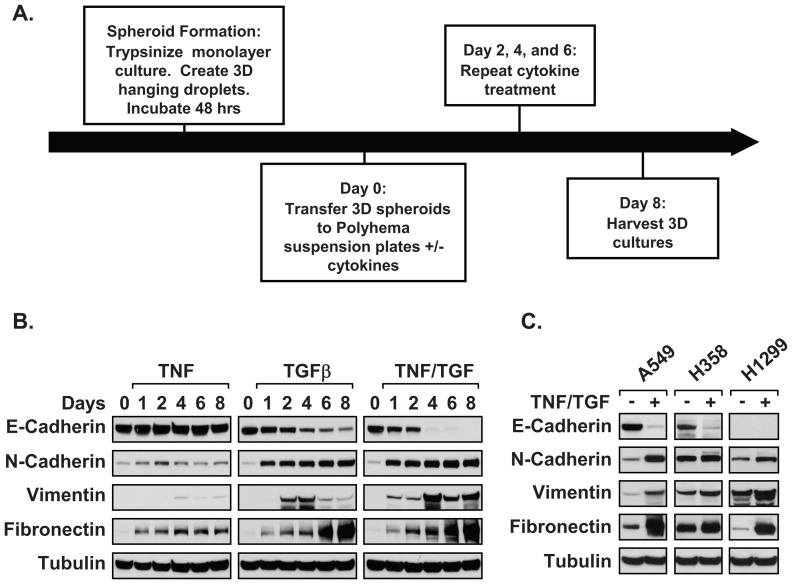
Establishment of three-dimensional multicellular culture model for EMT studies. (A) A timeline illustrates the procedure used to create a three-dimensional mesenchymal cell population from confluent monolayers. (B) Spheroid cultures of A549 cells were treated, with TNF, TGFβ, or both cytokines every forty-eight hours for the indicated times. Immunoblot analysis measured changes in epithelial (E-cadherin) and mesenchymal (N-cadherin, Vimentin, and fibronectin) markers over an eight day timecourse. (C) 3D cultures of multiple NSCLC cell lines (A549, H358, H1299) were incubated for ninety-six hours in the absence or presence of TNF and TGFβ. Epithelial and mesenchymal markers were subsequently measured by immunoblot. Results from Figure 1B and 1C are representative examples from at least three independent experiments; α-tubulin acts as a protein loading control.

TNF has been shown to potentiate TGFβ-mediated EMT through the activation of co-stimulatory pathways [15]. To confirm this observation in our three-dimensional (3D) model, a timecourse was performed using both cytokines in tandem and alone. Multicellular spheroid cultures were created using a modified hanging droplet method [36]. After two days, spheroids were suspended in poly-HEMA coated plates and treated every two days with the indicated cytokines to induce EMT (Figure 1A). Samples were collected from untreated (0 days) and cytokine-treated cultures (1–8 days). Epithelial (E-cadherin) and mesenchymal (N-cadherin, Vimentin, and Fibronectin) markers were measured by immunoblot. Treatment with TNF resulted in a modest increase in N-cadherin and Fibronectin, but failed to show differences in other markers (Figure 1B). Consistent with the induction of EMT, TGFβ treatment resulted in a loss of E-cadherin expression and an increase in N-cadherin, Vimentin, and Fibronectin. Moreover, co-stimulation with TNF and TGFβ yielded a more mesenchymal phenotype and persisted throughout the eight day time course (Figure 1B). Importantly, stimulation with TNF and TGFβ effectively induced EMT in both A549 and H358 cell lines within four days of treatment, compared to H1299, which already shows changes in E-cadherin and vimentin (Figure 1C). Based on results in Figure 1, we used the four day timeframe throughout our remaining experiments.

### 3D cultures undergo EMT more efficiently than 2D cultures

**Figure 2:**
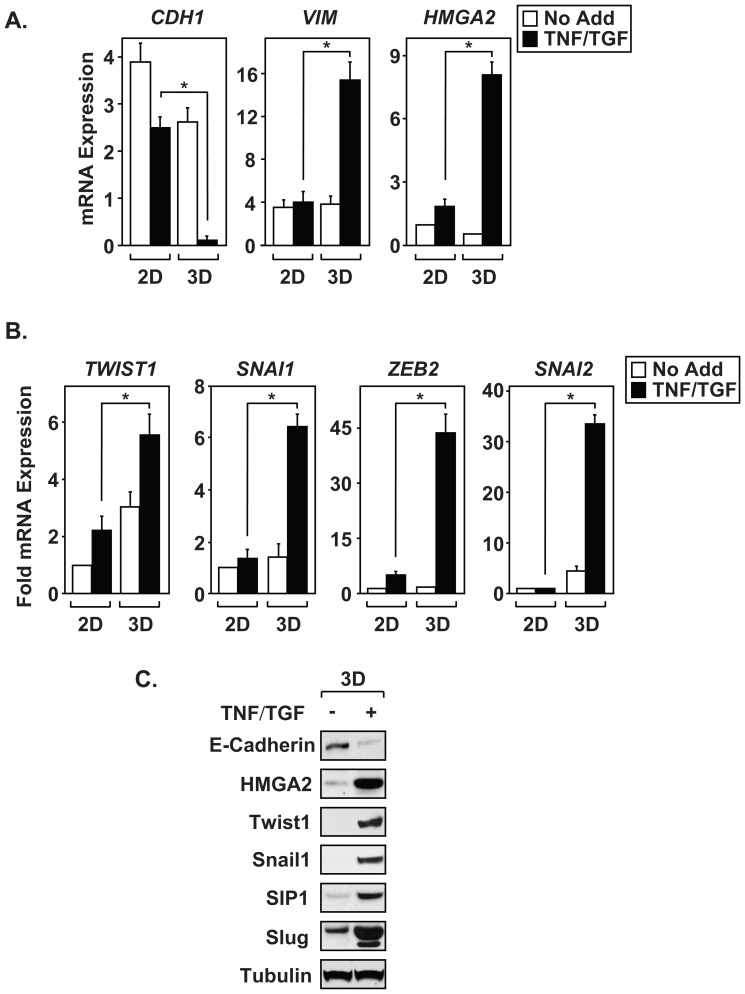
Three-dimensional cultures show enhanced sensitivity to cytokine treatment. (A and B) Monolayer (2D) and 3D cultures of A549 cells were left alone (No Add) or treated with TNF and TGFβ (TNF/TGF) for ninety-six hours. Expression of epithelial markers (*CDH1*), mesenchymal markers (*VIM, HMGA2*), and EMT master-switch transcription factors (*TWIST1, SNAI1, ZEB2, SNAI2*) were measured by QRT-PCR. (C) Immunoblot analysis of 3D A549 cultures, left alone (No Add) or treated with TNF and TGFβ (TNF/TGF), was performed on E-cadherin, Vimentin, HMGA2, Twist1, Snail1, Sip1, Slug, and α-tubulin. Results in Figure 2A and 2B were normalized to *GAPDH*, and are calculated mean ± S.D, *p<0.05, N = 3. Immunoblots in Figure 2C are representative example from at least three independent experiments.

To determine whether 3D A549 cultures undergo EMT more efficiently than two-dimensional (2D) monolayer cultures, we measured expression of epithelial and mesenchymal markers in response to stimulation with TNF and TGFβ as described in Figure 1. Following cytokine treatment, 3D cultures show significant loss of *CDH1/E-cadherin* expression when compared to 2D cultures (Figure 2A). Moreover, the spheroids also possess increased expression of mesenchymal markers *VIM*, *HMGA2*, and the EMT master-switch transcription factors, *TWIST1*, *SNAI1/Snail1*, *SNAI2/Slug* and *ZEB2/Sip1* (Figures 2A and 2B). Immunoblot analysis of spheroid cultures confirm that the differential mRNA expression resulted in a corresponding change in protein levels (Figure 2C). Additionally, we examined changes in cellular morphology and E-cadherin localization by microscopy. Both 2D and 3D cultures were treated with cytokines as described, trypsinized, re-plated on glass coverslips, and indirect immunofluorescent staining was carried out eighteen hours later. As expected, untreated monolayer and spheroid A549 samples showed robust E-cadherin expression, though the junctional localization appeared diminished in cells from the 3D cultures (Figure S1). Furthermore, cells derived from cytokine-treated spheroids displayed enhanced loss of E-cadherin when compared to 2D treated samples, suggesting that 3D cultures underwent more efficient EMT. Results shown in Figure 2 and Figure S1 illustrate significant EMT induction in 3D cultures as measured by changes in mesenchymal markers, EMT master-switch transcription factor expression, and cellular morphology.

### Mesenchymal NSCLC cells are invasive and endogenously express genes known to promote stem-like properties

**Figure 3:**
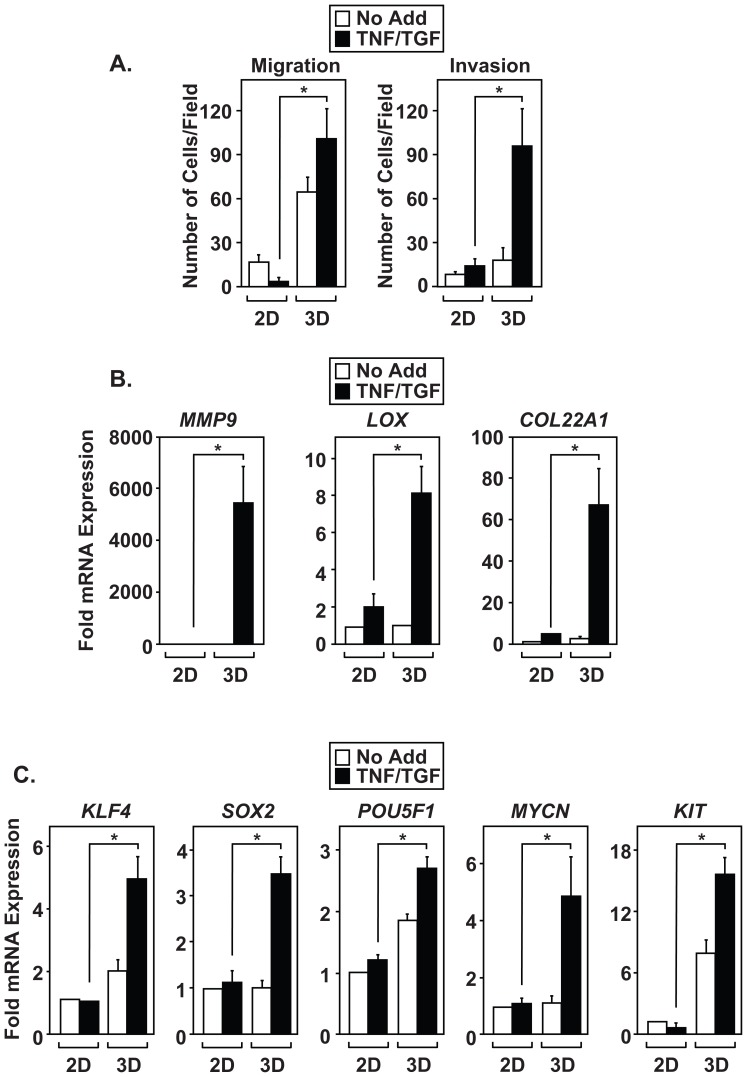
Efficient induction of EMT promotes invasion and the expression of genes required to maintain CICs. Monolayer and 3D A549 cultures were left alone (No Add) or treated with TNF and TGFβ (TNF/TGF) for ninety-six hours. (A) Cells were disaggregated and subsequently subjected to migration and invasion assays. (B and C) Expression of invasion (*MMP-9, LOX, COL22A1*) and stem cell markers (*KLF4, Sox2, POU5F1, MYCN, and KIT*) was measured by QRT-PCR. Results in Figure 3 are calculated mean ± S.D, *p<0.05, N = 3. Results from 3B and 3C were normalized to *GAPDH*.

Phenotypically, mesenchymal cells have high migration rates and secrete enzymes that degrade extracellular matrix to facilitate cellular invasion. Using *in vitro* transwell assays, we measured the migration and invasion characteristics of A549 cells grown as either 2D or 3D cultures. Interestingly, untreated 3D spheroid cultures showed higher migration rates than 2D monolayer cultures (Figure 3A, left). However, treatment of 3D cultures with TNF and TGFβ further potentiated migration when compared to untreated 3D cultures. Spheroids treated with cytokines invaded through Matrigel more effectively than any other condition (Figure 3A, right). Additionally, cytokine treated A549 spheroids displayed upregulated expression of *MMP9*, *LOX*, and *COL22A1* (Figure 3B), genes known to potentiate invasion [41], [42]. These results demonstrate that culturing 3D spheroids in the presence of TNF and TGFβ establishes a highly invasive mesenchymal population. Finally, cytokine-treated spheroids showed endogenous upregulation of markers associated with de-differentiation and maintenance of CICs [43]–[48], including *KLF4, SOX2, POU5F1/Oct4, MYCN*, and *KIT* (Figure 3C). Data shown in Figure 3 indicate that co-stimulation of spheroids with TNF and TGFβ promotes phenotypic changes in A549 cells that result in increased invasion and expression of gene products associated with stem-like properties.

### Mesenchymal cells are highly metastatic and display cancer initiating phenotypes

**Figure 4:**
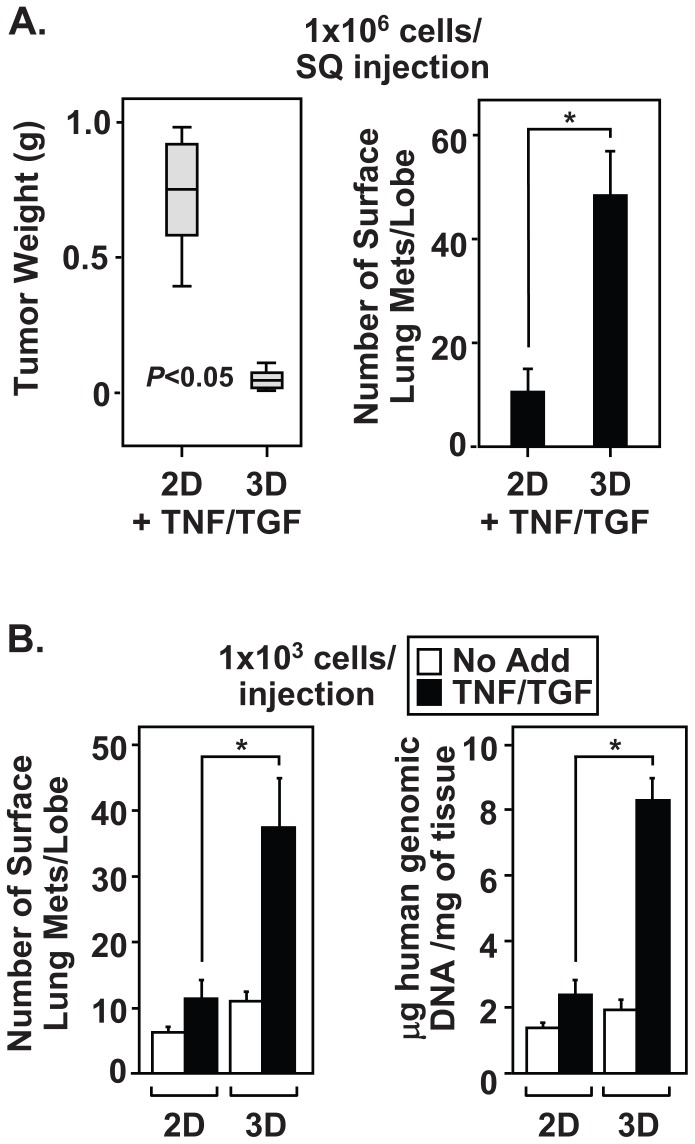
Cytokine-treated 3D cultures contain CICs with increased metastatic potential. (A) Monolayer and 3D A549 cultures were treated with TNF and TGFβ for ninety-six hours. Cells were disaggregated and SC injected into nude mice (1×10^6^ cells/animal). Forty days later, the primary SC tumors were resected and weighed. Additionally, the lungs were excised and the number of surface metastases were determine. (B) Monolayer and 3D A549 cultures were either left untreated or treated with TNF and TGFβ and limiting cell numbers (1×10^3^/animal) were SC injected into nude mice to evaluate the presence of CICs. Metastasis was evaluated by surface lung tumor count and lung tumor burden was evaluated using genomic QRT-PCR to detect human DNA in total lung tissue. Weight and lung metastases data from Figure 4 are mean ± S.D. of five mice per condition, *p<0.05, N = 3 independent experiments. Genomic QRT-PCR data from Figure 4B are normalized to total lung tissue (mg).

To examine whether induction of EMT promotes the development of CICs *in vivo*, we utilized a xenograft tumor model in nude mice. TNF and TGFβ treated 2D and 3D cultures were disaggregated and cell suspensions were SC injected into the right flank of nude mice. Forty days later, animals were sacrificed and SC tumors were resected and weighed while the lungs were excised and scored for surface metastases. To our surprise, TNF and TGFβ-treated cells did not form SC tumors to the same extent as cytokine-treated 2D cultures (Figure 4A, left). However, examination of the lung surface in these mice revealed extensive metastasis (Figure 4A, right). The only plausible explanation for these results is that mesenchymal cells from 3D cultures invaded and metastasized to the lung without developing SC tumors.

Measuring the extent of metastasis under limiting cell dilution proves a reliable test for the presence of enriched CICs in epithelial-derived tumors [49]. Therefore, experiments were repeated using one-thousand cells per SC injection. Cell suspensions, derived from TNF and TGFβ treated spheroids, produced more surface lung metastases under limiting cell dilution than cytokine-treated monolayers or untreated 3D cultures (Figure 4B left). Limiting cell dilution assays indicate that induction of EMT in 3D cultures produces a CIC population that effectively metastasizes to lung. As expected, analysis of DNA isolated from mouse lungs confirmed the presence of metastatic burden and verified that the lesions were of human origin (Figure 4B right). We conclude from the experiments in Figure 4 that de-differentiation, CIC formation, and metastatic potential are all significantly enhanced in EMT-induced spheroid cultures.

### NF-κB is constitutively active in 3D cultures and is required for induction of EMT

**Figure 5:**
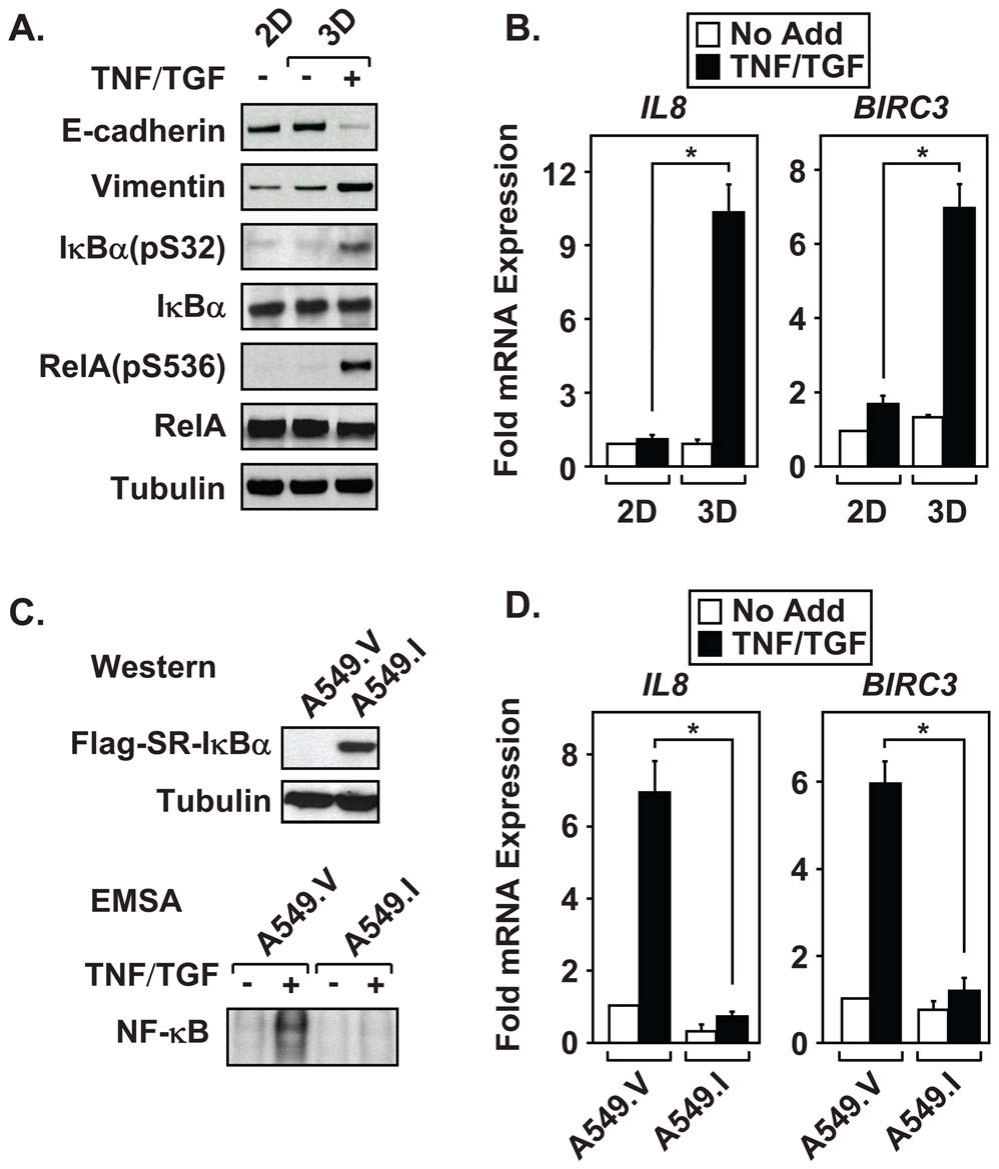
Mesenchymal cells display constitutive NF-κB activity. Monolayer and 3D cultures of A549 cells were incubated with cytokines for ninety-six hours. (A) Mesenchymal A549 cells display constitutive NF-κB activated pathways, as determined using phospho-specific antibodies to IκBα and RelA. (B) Untreated and TNF and TGFβ stimulated 2D and 3D cultures of A549 cells were harvested and analyzed for expression of NF-κB regulated genes by QRT-PCR. (C and D) Three dimensional cultures of A549.V (vector control) and A549.I (SR-IκB) were incubated for ninety-six hours in the absence or presence of TNF and TGFβ. (C) Immunoblots confirm the expression of the Flag-tagged SR-IκBα in the A549.I line, which successfully blocked nuclear translocation and DNA binding, as measured by EMSA. (D) QRT-PCR confirmed the inability of A549.I cell to upregulate NF-κB-regulated genes following TNF and TGFβ treatment. Immunoblots in Figure 5A are a representative example from three independent experiments. Results in Figure 5B and 5D are calculated mean ± S.D, *p<0.05, N = 3. RNA values were normalized to *GAPDH*.

TNF, a potent NF-κB activator, enhances induction of EMT in NSCLC cell lines. Therefore, we assessed whether EMT induction results in activation of NF-κB signaling by immunoblot. Interestingly, mesenchymal A549 spheroids displayed constitutive IKK activity as measured by phospho-specific antibodies that detect IκBα pS32 and RelA pS536 (Figure 5A and Figure S2A). Change in E-cadherin and Vimentin levels confirmed efficient EMT in the cytokine-treated spheroids. Moreover, QRT-PCR experiments demonstrated increased expression of NF-κB-regulated genes *IL8* and *BIRC3/cIAP2* in mesenchymal 3D cultures (Figure 5B). Collectively, these data indicate that cytokine-treatment of 3D A549 cultures results in the increased phosphorylation of IKK-regulated substrates and constitutive NF-κB transcriptional activation.

To determine the importance of NF-κB activity during induction of EMT in NSCLC cell lines, stable clonal pools expressing the super-repressor IκBα (SR-IκBα) were generated. The SR-IκBα is resistant to proteasomal degradation, and consequently sequesters NF-κB in the cytosol. Cells expressing the SR-IκBα protein therefore display an inhibition of NF-κB-mediated transcription [50]. Figure 5C (top) confirms expression of Flag-tagged SR-IκBα in A549 stable cells (A549.I) compared to empty vector control cells (A549.V). Furthermore, nuclear protein extracts from A549.I spheroid cultures, treated with TNF and TGFβ, lacked NF-κB DNA binding activity as compared to A549.V extracts (Figure 5C, bottom). Supershift experiments confirm that the NF-κB activity is composed predominantly of a RelA-p50 heterodimer complex (Figure S2B). QRT-PCR assays show repressed cytokine-mediated induction of *IL8* and *BIRC3/cIAP2* in A549.I cells when compared to control cells A549.V (Figure 5D). In contrast to high doses of TNF (100 ng/ml), low doses (10 ng/ml) did not result in a loss of cell viability in A549.I lines, since expression of the house keeping gene, HPRT, did not change and was used for normalization in Figure 5D. These data verify that SR-IκBα expression in the A549.I cell line effectively blocks NF-κB transcriptional activity.

### Characterization of NF-κB in potentiating the mesenchymal phenotype

**Figure 6:**
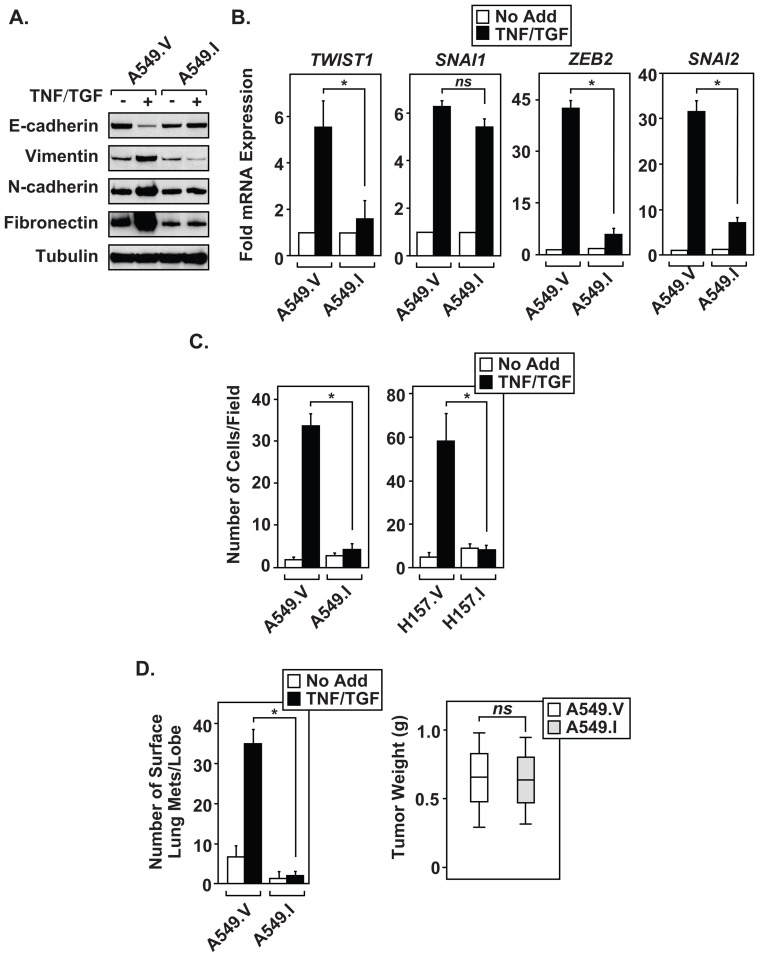
NF-κB is required for the maintenance of CICs and lung metastasis. (A) A549.I cells fail to show changes in mesenchymal markers, as determined by immunoblot analysis. (B) NF-κB is required to upregulate mRNA expression of master-switch transcription factors. (C) Spheroid cultures of A549 and H157 cell lines, expressing empty vector or the Flag-IκB super-repressor, were left alone (No Add) or treated with TNF and TGFβ (TNF/TGF) for ninety-six hours. The cells were disaggregated and subjected to invasion assays. (D) A549.V and A549.I 3D cultures were left alone (No Add) or treated with TNF and TGFβ (TNF/TGF) for ninety-six hours. The cells were disaggregated and SC injected into nude mice (1×10^6^/animal). Forty days later, animals were sacrificed and the number of surface lung metastasis were determined. In addition, SC tumors were excised and wet tumor weight determined. Weight and lung metastases data from Figure 6 are mean ± S.D. of five mice per condition, *p<0.05, N = 3 independent experiments. The graphs in Figure 6 are mean ± S.D., *p<0.05, of three independent experiments. Data with *P* values greater than 0.05 were considered not significant (*ns*). QRT-PCR experiments are normalized to *GAPDH* expression.

NF-κB has been shown to regulate the expression of EMT master-switch transcription factors in multiple model systems [21]–[24]. Therefore, we hypothesized that inhibiting NF-κB activity in the A549.I cell line would dampen EMT induction. Immunoblot analysis confirmed that A549.I cells fail to down regulate E-cadherin expression or upregulate mesenchymal markers (Vimentin, N-cadherin and Fibronectin) compared to control cells (Figure 6A). Moreover, cytokine-treated A549.I cells showed only minimal upregulation of *TWIST1*, *ZEB2* and *SNAI2* gene expression following TNF and TGFβ treatment (Figure 6B). These results indicate that NF-κB is required to upregulate *TWIST1*, *ZEB2* and *SNAI2*, while expression of *SNAI1* appears independent of NF-κB-dependent transcription in the A549.I cell line. These results suggest that the expression of critical EMT master-switch transcription factors requires NF-κB activity.

Next, we assessed whether NSCLC required NF-κB for invasion using transwell assays. Inhibited NF-κB activity in A549.I cells abolished invasion through Matrigel when compared to the control lines (Figure 6C). This effect was not cell-line specific since another NSCLC line expressing the SR-IκB (H157.I) showed similar results as A549.I cells. Because data shown in Figure 6 indicate that NF-κB is required for NSCLC to undergo EMT, we tested the A549.V and A549.I cell for their ability to metastasize to lung using a nude mouse model. As expected, cytokine-treated A549.I cells failed to form lung metastases (Figure 6D, left). The inability of these cells to metastasize to lung was not due to a loss of cell viability or an inability to form primary tumors, since untreated A549.I formed SC tumors with similar growth rates as A549.V cells (Figure 6D, right). Thus, data shown in Figure 6 indicates that TNF and TGFβ treated 3D NSCLC cultures require NF-κB to upregulate master-switch transcription factors, induce EMT, and promote invasive properties. Moreover, without NF-κB transcriptional activity A549 cells lose their ability to metastasize to lung without impacting primary tumor growth.

## Discusson

### NF-κB regulates EMT to potentiate metastatic progression of NSCLC

We implemented a simple and relatively quick 3D culture system to examine the importance of NF-κB signaling during EMT induction and CIC propagation within NSCLC cell lines. In response to TNF and TGFβ exposure, A549 spheroid cultures displayed a loss of E-Cadherin and elevated expression of mesenchymal markers, N-Cadherin, Vimentin, and Fibronectin. The increased expression of mesenchymal protein markers likely occurs due to induction of the EMT master-switch transcription factors, *TWIST1*, *SNAI1*, *SNAI2* and *ZEB2*. Furthermore, spheroid populations of mesenchymal A549 cells show elevated expression of endogenous transcription factors known to potentiate dedifferentiation, including *KLF4*, *SOX2*, *POU5F1*, *MYCN* and *KIT*. Interestingly, mesenchymal A549 cells from spheroid cultures failed to generate large SC tumors, compared to 2D cultures. Despite this effect, cytokine-treated 3D A549 cells displayed elevated lung surface metastatic lesions. These results support the hypothesis that CICs extravasated into the circulatory system and metastasize to the lung without forming SC tumors. We further demonstrated that EMT-induced A549 3D cultures effectively metastasize to lung under limiting cell dilutions, confirming the presence of an enriched “stem-like” CIC population. Thus, our results suggest that EMT induction effectively selects for self-renewing CIC with metastatic potential, a phenotype described by Dieter and colleagues as self-renewing long-term tumor initiating cells responsible for color cancer metastasis [51]. Since IKK and NF-κB pathways have been linked to EMT and development of CICs [21]–[25], [31], we examined whether mesenchymal A549 cells upregulate NF-κB transcriptional activity. Surprisingly, EMT-induced spheroid A549 cultures displayed chronic IKK activity as measured by phosphorylation of IκBα(pS32) and RelA(pS536), and by constitutive expression of *IL8* and *BIRC3* transcripts. Moreover, cytokine-treated spheroid A549 cultures maintained the activation of IKK signaling pathways well beyond the half-life of the TNF and TGFβ cytokines added to the culture media. These results suggest that mesenchymal A549 spheroid cultures must produce autocrine factors capable of maintaining the EMT phenotype. Importantly, constitutive NF-κB activity proves essential for effective EMT partially through its ability to upregulate the master-switch transcription factors *TWIST1*, *ZEB2* and *SNAI2*. As a result, the loss of NF-κB activity prohibited cytokine-treated spheroid A549 cells from becoming invasive and also abolished lung metastasis in the mouse xenograft model. This work firmly establishes a role for NF-κB in the induction of EMT and for the development of NSCLC CICs that promote metastasis.

### Spheroid models and the propagation of CICs

Various 3D culture models have been developed that more accurately mimic tumor biology, such as cell-cell contacts, extracellular matrix composition, and nutrient access/gradients [52]–[54]. The advantage to using the hanging drop technique, over other techniques, is that 2D cultures can be quickly expanded to form multicellular aggregates that share similar size and shape, and mesenchymal populations are generated within six days. Data provided in Figure 1C demonstrate that multiple NSCLC cell lines form compact spheroids that undergo highly reproducible EMT when exposed to TNF and TGFβ. Moreover, these spheroids possess increased sensitivity to TNF and TGFβ compared to monolayer cultures (Figures 2, 3, and 4). Therefore, we utilized this 3D system to examine EMT and CIC formation in NSCLC cell lines. Surprisingly, A549 spheroids show increased migration without requiring exposure to TNF and TGFβ and despite expressing epithelial markers (Figure 1B, 2A, and 3A). This indicates that phenotypic changes occur in 3D cultures prior to exposure to EMT-inducing cytokines. However, increased invasion is restricted to cytokine-induced A549 spheroid cultures and corresponds with the upregulation of matrix and extracellular remodeling enzymes known to induce invasive properties [41], [42]. Therefore, spheroid cultures are poised to respond to TNF and TGFβ cytokines and are able to sustain EMT reprogramming. Together, we establish that CIC populations formed from EMT induction of 3D NSCLC cell lines provide a useful tool for further characterization of cancer progression in the lung.

### Mesenchymal A549 cells show constitutively active NF-κB signaling pathways

Constitutive NF-κB activation occurs in many different types of hematopoietic and epithelial-derived carcinomas. However, mutations that result in chronic activation of NF-κB signaling are extremely rare in epithelial cancers [19]. Thus, activation of NF-κB most likely results from autocrine and paracrine signaling within the tumor microenvironment rather than genetic alterations [2], [19]. Our data support this hypothesis by showing that TNF- and TGFβ-treated A549 spheroid populations both undergo EMT and maintain constitutive NF-κB signaling (Figure 5A and 5B). Rather than using co-culture systems, which introduce contaminating cell types other than NSCLC cells, we chose to treat A549 spheroids with EMT-inducing cytokines. TNF and TGFβ were selected because within the tumor microenvironment, TNF is believed to be produced predominantly by tumor-associated macrophages, while TGFβ is secreted by fibroblast and endothelial cells. This combination of cytokines not only effectively and reproducibly induces EMT, but also facilitates a reprogramming event that results in chronic NF-κB signaling. The molecular mechanism by which this occurs is currently unknown, but most likely is due to an increase in NF-κB-regulated gene products that function in an autocrine-dependent manner to maintain active NF-κB.

### Inflammatory regulatory circuits that drive constitutive NF-κB activation

In the past two years, evidence has emerged that an epigenetic switch occurs during breast cancer transformation in which inflammatory circuits involving IL6 and IL8 mediate self-renewal of CICs [55]–[57]. Ginesteir and colleagues showed that breast CICs upregulate the IL8 receptor CXCR1 to potentiate self-renewal, tumorigenicity and metastasis [57]. Additional studies indicate that oncogenic transformation of breast cancer cells leads to chronic activation of NF-κB required to upregulate Lin-28B and downregulate the negative microRNA regulator of IL6, Let-7a [56]. As a result, IL6 provides an inflammatory feedback loop that further activates NF-κB as well as the STAT3 signaling pathway [56], [58]. Interestingly, this pro-inflammatory feedback loop also exists in some prostate and hepatocellular carcinomas, but only a subset of lung cancers showed increased IL6 expression [56]. In addition to the autocrine feedback mechanism, IL6 signaling pathways downregulate mir200c in a chemically-induced transformed breast cancer cell line. Loss of mir200c subsequently results in constitutively activated NF-κB through an inflammatory feedforward signaling circuit [59]. In these papers [55], [56], [59], IL6 was found to be required for the maintenance of breast CICs.

Additional work is needed to determine the importance of IL8 and IL6 as feedforward mediators of NF-κB activation in mesenchymal NSCLC cell lines. As shown in Figure 5B, *IL8* is highly upregulated and maintained in mesenchymal A549 cultures; however, *IL6* transcripts do not significantly change between untreated 3D and cytokine-treated 3D cultures (Figure S2C). Thus, in agreement with IIiopoulos and colleagues [59], IL6 may not be a common requirement for CICs in lung cancer. However, since CXCR1 is highly expressed in A549 cells following exposure to DNA methytransferase inhibitors [60], inflammatory circuits that regulate promoter demethylation, as observed for IL6 signaling [59], may play an important role for controlling the IL8/CXCR1 responsiveness in lung cancers. Future work is needed to explore the importance of IL8/CXCR1 in the maintenance of constitutive NF-κB activation and development of NSCLC CICs.

## Supporting Information

Figure S1 **Cytokine-treated 3D A549 cells show increased fibroid and mesenchymal morphology.** Monolayer (2D) and 3D A549 cultures were left alone or treated with TNF and TGFβ for ninety-six hours. Cells were subsequently disaggregated, replated on glass coverslips, and cultured for an additional eighteen hours in 2% FBS. The cells were then fixed in methanol, and indirect immunofluorescence was used to detect the presence of junctional E-cadherin. Images are a representative field from three independent experiments.
(TIF)

Figure S2 **TNF and TGFβ-treated 3D A549 cells show increased RelA phosphorylation and nuclear DNA binding activity.** (A) Immunoblot analysis of 3D A549 cells indicates that cells display constitutive RelA phosphorylation upon co-stimulation with both TNF and TGFβ over the three day period. (B) Nuclear extracts from cytokine-treated 3D control A549.V cells show elevated NF-κB binding activity by EMSA, compared to unstimulated cell extracts. The NF-κB DNA-protein complex is composed of both RelA and p50 proteins as detected by antibody super shift (SS) assays. (C) In contrast to *IL8* expression shown in Figure 5B, cytokine-treated 3D cultures fail to upregulate *IL6* transcripts as measured by QRT-PCR.
(TIF)

Table S1 **QRT-PCR Primers.**
(DOC)
