# Supplementary figures and images for: NF-κB Regulates Mesenchymal Transition for the Induction of Non-Small Cell Lung Cancer Initiating Cells
Source: PLoS One. 2013 Jul 30;8(7):e68597. doi: 10.1371/journal.pone.0068597 (PMC3728367; doi:10.1371/journal.pone.0068597)

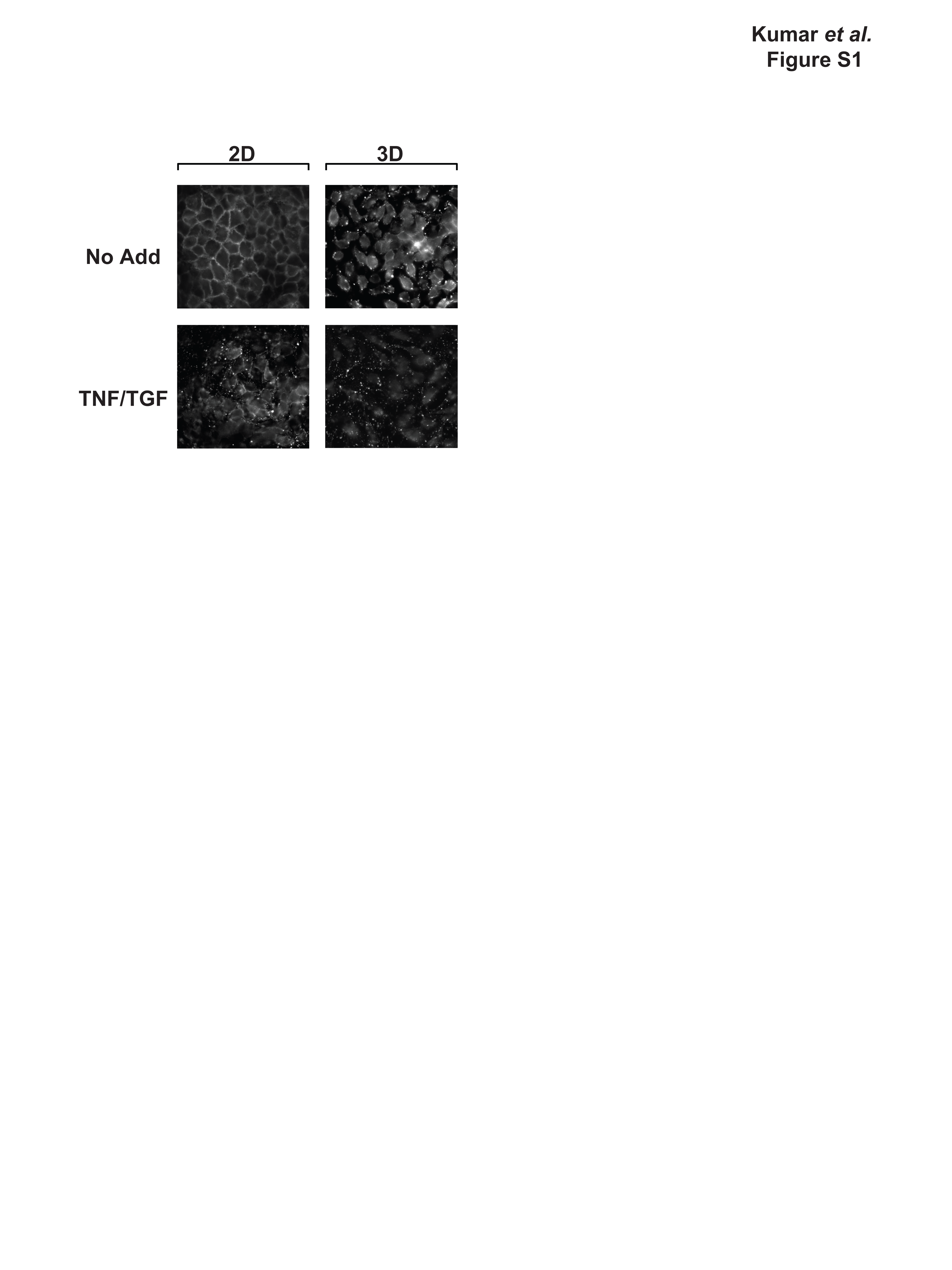

Supplement: Figure S1 — Cytokine-treated 3D A549 cells show increased fibroid and mesenchymal morphology. Monolayer (2D) and 3D A549 cultures were left alone or treated with TNF and TGFβ for ninety-six hours. Cells were subsequently disaggregated, replated on glass coverslips, and cultured for an additional eighteen hours in 2% FBS. The cells were then fixed in methanol, and indirect immunofluorescence was used to detect the presence of junctional E-cadherin. Images are a representative field from three independent experiments. (TIF) [file pone.0068597.s001.tif]

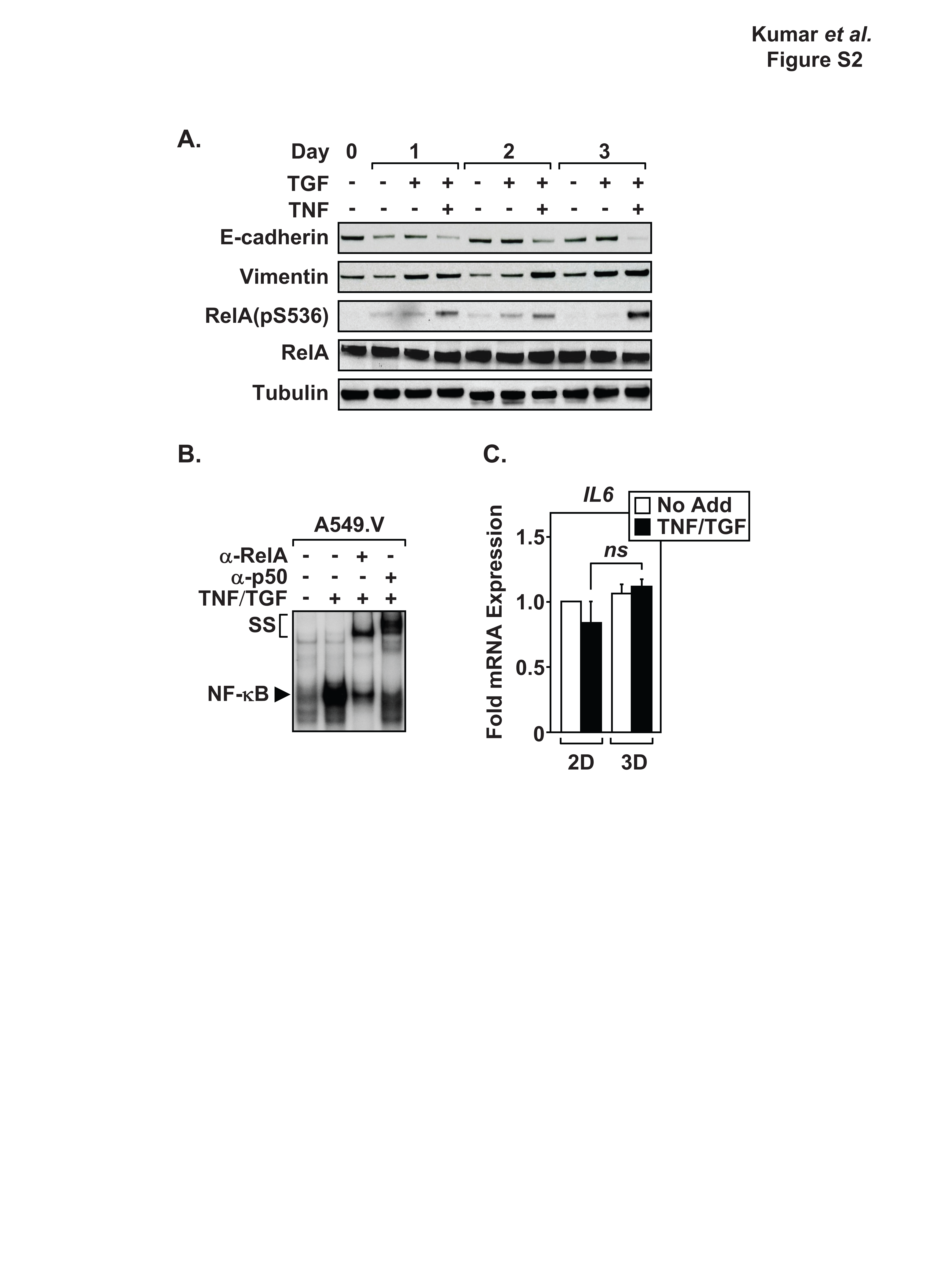

Supplement: Figure S2 — TNF and TGFβ-treated 3D A549 cells show increased RelA phosphorylation and nuclear DNA binding activity. (A) Immunoblot analysis of 3D A549 cells indicates that cells display constitutive RelA phosphorylation upon co-stimulation with both TNF and TGFβ over the three day period. (B) Nuclear extracts from cytokine-treated 3D control A549.V cells show elevated NF-κB binding activity by EMSA, compared to unstimulated cell extracts. The NF-κB DNA-protein complex is composed of both RelA and p50 proteins as detected by antibody super shift (SS) assays. (C) In contrast to IL8 expression shown in Figure 5B, cytokine-treated 3D cultures fail to upregulate IL6 transcripts as measured by QRT-PCR. (TIF) [file pone.0068597.s002.tif]
