# Supplementary material for: NF-κB Regulates Mesenchymal Transition for the Induction of Non-Small Cell Lung Cancer Initiating Cells
Source: PLoS One. 2013 Jul 30;8(7):e68597. doi: 10.1371/journal.pone.0068597 (PMC3728367; doi:10.1371/journal.pone.0068597)
Supplement: Table S1 — QRT-PCR Primers. (DOC) [file pone.0068597.s003.doc]

**SUPPLEMENTAL TABLE 1: QRT-PCR Primers**

| Primer | Sequence |
| --- | --- |
| *H. sapiens* *BIRC3* F  *H. sapiens* *BIRC3* R  *H. sapiens CDH1* F | 5’ – GCAGCAACCTCATTCAGAAA – 3’  5’ – GCAATGTCATCTGTGGGAAG – 3’  5’ – TGAGTGTCCCCCGGTATCTTC – 3’ |
| *H. sapiens CDH1* R | 5’ – CAGTATCAGCCGCTTTCAGATTTT – 3’ |
| *H. sapiens* *COL22A1* F | 5’ – ACGAACGCTAGGACAGAGCA – 3’ |
| *H. sapiens* *COL22A1* R | 5’ – GTCTTCCACGTGTCCGACTT – 3’ |
| *H. sapiens* *GAPDH* F | 5’ – GAAGGTGAAGGTCGGAGTC – 3’ |
| *H. sapiens* *GAPDH* R | 5’ – GAAGATGGTGATGGGATTTC – 3’ |
| *H. sapiens* *HMGA2* F | 5’ – AGTCCCTCTAAAGCAGCTCAAAAG – 3’ |
| *H. sapiens* *HMGA2* R  *H. sapiens IL6* F  *H. sapiens IL6* R | 5’ – GCCATTTCCTAGGTCTGCCTC – 3’  5’ – TAGCCGCCCCACACAGACAG – 3’  5’ – GGGTTGGTGTTTACGGTCGG – 3’ |
| *H. sapiens IL8* F | 5’ – CTCTTGGCAGCCTTCCTG – 3’ |
| *H. sapiens IL8* R | 5’ – CTGTGTTGGCGCAGTGTG – 3’ |
| *H. sapiens KIT* F | 5’ – AGGATTCCCAGAGCCCACAATAG – 3’ |
| *H. sapiens KIT* R | 5’ – ACGGTGGCCCAGATGAGTTTAG – 3’ |
| *H. sapiens KLF4* F | 5’ – ACCAGGCACTACCGTAAACACA – 3’ |
| *H. sapiens KLF4* R | 5’ – GGTCCGACCTGGAAAATGCT – 3’ |
| *H. sapiens LOX* F | 5’ – CGGCGGAGGAAAACTGTCT – 3’ |
| *H. sapiens LOX* R | 5’ – TGAGCAGCACCCTGTGATCA – 3’ |
| *H. sapiens MMP9* F | 5’ – TTGACAGCGACAAGAAGTGG – 3’ |
| *H. sapiens MMP9* R | 5’ – GCCATTCACGTCGTCCTTAT – 3’ |
| *H. sapiens MYCN* F | 5’ – CGCAAAAGCCACCTCTCATTA – 3’ |
| *H. sapiens MYCN* R | 5’ – TCCAGCAGATGCCACATAAGG – 3’ |
| *H. sapiens POU5F1* F | 5’ – ACCCCTGGTGCCGTGAAGC – 3’ |
| *H. sapiens POU5F1* R | 5’ – CAGATGGTCGTTTGGCTGAATACC – 3’ |
| *H. sapiens SNAI1* F | 5’ – CACTATGCCGCGCTCTTTC – 3’ |
| *H. sapiens SNAI1* R | 5’ – GGTCGTAGGGCTGCTGGAA – 3’ |
| *H. sapiens SNAI2* F | 5’ – ATGAGGAATCTGGCTGCTGT – 3’ |
| *H. sapiens SNAI2* R | 5’ – CAGGAGAAAATGCCTTTGGA – 3’ |
| *H. sapiens SOX2* F | 5’ – GCCCAGGAGAACCCCAAGATG – 3’ |
| *H. sapiens SOX2* R | 5’ – GCTCGCAGCCGCTTAGCCTC – 3’ |
| *H. sapiens TWIST1* F | 5’ – CGGGAGTCCGCAGTCTTA – 3’ |
| *H. sapiensTWIST1* R | 5’ – CTTGAGGGTCTGAATCTTGCT – 3’ |
| *H. sapiens VIM* F | 5’ – CTTCGTGAATACCAAGACCTGC – 3’ |
| *H. sapiens VIM* R | 5’ – TATCAACCAGAGGGAGTGAATCC – 3’ |
| *H. sapiens ZEB2* F | 5’ – CAATACCGTCATCCTCAGCA – 3’ |
| *H. sapiens ZEB2* R | 5’ – CCAATCCCAGGAGGAAAAAC – 3’ |
| *ERV3* F | 5’ – ATGGGAAGCAAGGGAACTAAT – 3’ |
| *ERV3* R | 5’ – CCCAGCGAGCAATACAGAATTT – 3’ |
| F, forward primer; R, reverse primer. | |
